# Renaissance of glucocorticoids in critical care in the era of COVID-19: ten urging questions

**DOI:** 10.1186/s13054-022-04185-9

**Published:** 2022-10-08

**Authors:** Martin S. Winkler, Marcin F. Osuchowski, Didier Payen, Antoni Torres, Steffen Dickel, Tomasz Skirecki

**Affiliations:** 1 Department of Anaesthesiology and Intensive Care Medicine, University Medical Center, Georg-August University of Göttingen, Robert-Koch-Str. 40, 37075 Göttingen, Germany; 2 Ludwig Boltzmann Institute for Traumatology Ludwig Boltzmann Institute for Trauma in Cooperation with the AUVA, Vienna, Austria; 3 Emeritus Professor of Anesthesiology and Critical Care, University of Paris 7, Cité, Sorbonne, Paris, France; 4 Servei de Pneumologia, Hospital Clinic IDIBAPS, Universitat de Barcelona, Centro de Investigación Biomedica En Red-Enfermedades Respiratorias, Instituto de Salud Carlos III, Madrid, Spain; 5 Department of Translational Immunology and Experimental Intensive Care, Centre of Postgraduate Medical Education, Marymoncka 99/103, 01-813 Warsaw, Poland

## Abstract

The 40-year-old experience with glucocorticosteroids (GCs) in the context of severe infections is complex and troublesome. Recently, however, a clear indication for GCs in severe COVID-19 has been established. This may constitute a harbinger of a wider use of GCs in critical illnesses. A fundamental prerequisite of such an action is a better understanding of the heterogeneity of critical illness and GCs operationalization within the precision medicine approach. In this perspective, we formulate ten major questions regarding the use of GCs in critical illness. Answering them will likely facilitate a new era of effective and personalized GCs use in modern critical care.

The application of glucocorticoids (GCs) in sepsis has been discussed for decades yet their use remains controversial. Findings from the RECOVERY trial confirmed by the REMAP-CAP have revived an interest in GCs as a current and effective standard-of-care (SoC) treatment of severe SARS-CoV-2 pneumonia (COVID-19) [1–3]. In contrast, the current sepsis guidelines feature only a weak recommendation for GCs reflecting a poor mechanistic understanding of GCs action and limited evidence regarding their therapeutic efficacy in sepsis [4]. In this perspective, we formulate ten urgent questions addressing the use of GCs in severe infections, answers to which will aid in improving the outcomes of critically ill patients (Fig. 1).

**Fig. 1:**
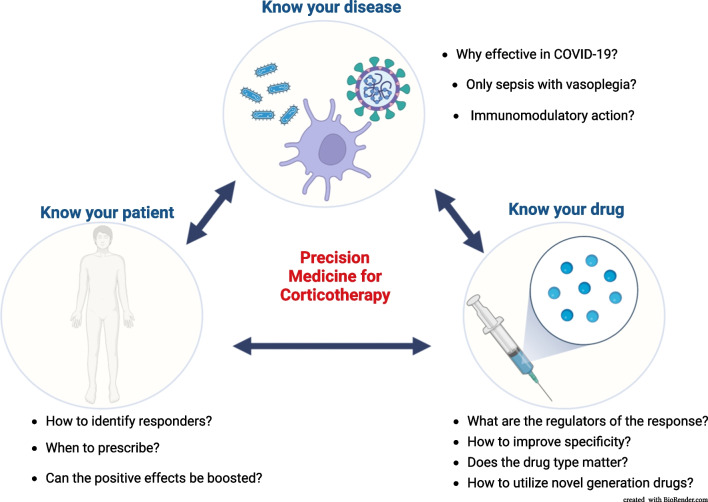
Ten emerging questions facilitating corticotherapy in the critical care

## Know your disease

### Why are GCs effective in COVID-19 but not in bacterial sepsis nor severe influenza?

There is a striking contrast of the efficacy of corticotherapy between COVID-19 [2], septic shock [4] and influenza [5]. Any conclusion regarding clinical improvements by GCs must be examined through a prism of specific end-point benefits such as reduction in mortality, increase in ventilatory-free days, improved hemodynamic stability, reduced intensity of fibrotic repair. GCs reduced 28-day mortality by 30% in mechanically ventilated patients, but not in patients without a respiratory support requirement [1]. When this endpoint is retrospectively applied to previous studies testing GCs in ARDS and (non-COVID-19) sepsis, the signal remains vague despite an adequate design of those trials. This provokes a subsequent question on why the benefit observed in COVID-19 was not also apparent in influenza-induced ARDS nor bacterial sepsis? To answer it, a clear picture of molecular mechanisms involved in COVID-19 is needed, also accounting for potential differences related to SARS-CoV-2 versus other types of infecting pathogens. Next, a longitudinal study of inflammatory response in presence/absence of GCs may clarify how GCs perform in COVID-19 and in other etiologies. The discrepancy in the efficacy of GCs between COVID-19 and influenza can be due to differences in: (1) the study design (e.g., administration timing/phase, dose, inclusion criteria of patients), (2) the nature/clinical course of COVID-19 infection versus influenza infection, and (3) the features of SARS-CoV-2 virus versus influenza viruses. In severe influenza, studies were typically non-randomized; only few RCTs tested high GC doses and they were frequently burdened by multiple confounding factors. The existing observational studies did not demonstrate any GC-dependent mortality reduction. Clear differences in the immune response to an infection with SARS-CoV-2 compared to influenza viruses were revealed and include: (1) weaker induction of chemokines, (2) strong activation of T-cells and distinct monocytes and (3) hyperactivated neutrophils response with a weaker direct epithelial injury by SARS-CoV-2 [6–8]. Severe COVID-19 is characterized by specific endothelial and immunothrombotic changes and patients who succumbed to COVID-19 presented with features of pulmonary fibrosis [9]. Although GCs are used in the treatment of exacerbations of idiopathic pulmonary fibrosis despite poor evidence of benefits [10], it is possible that corticotherapy is more effective in COVID-19-related fibrosis. This concept is supported by a small study demonstrating efficacy of a low-to-moderate corticotherapy in the fibrotic phase of critical COVID-19 but not the earlier alveolitis phase [11]. It remains unclear which particular aspect of COVID-19 pathogenesis is affected by GCs but each of the above-mentioned constitutes a potential target. Relatively low doses of beneficial corticotherapy and their low efficacy in alveolitis phase [11] suggest that immunosuppression may not be the only mechanism of GCs action. Benefits of a late corticotherapy initiation can be related to a paradoxical insensitivity of cells to GCs induced by viral infection [12]. Potential COVID-19-specific targets for GCs include also a direct interaction with viral proteins [13] and specific isoforms of p65 [14].

### Is refractory vasoplegia the only viable target for GCs treatment in septic shock?

In critically ill patients, the critical illness-related corticosteroid insufficiency (CIRCI) has been the key indication for corticotherapy [15]. CIRCI is described as an inadequate hormonal stress response for the severity of critical illness. Nevertheless, the role of GCs in septic shock remains unclear. Indeed, a 90-day mortality in septic shock patients was lower under corticotherapy in the APROCCHSS trial [16] among others, while some other large RCTs showed conflicting results including elevated mortality [17]. Summarizing, administration of GCs is recommended only in refractory septic shock without a synacthen stimulation test [4]. It is speculative that administration of GCs can even be harmful in milder sepsis manifestations. Since severe vasoplegia is difficult to treat, corticotherapy seems justifiable when the risks are weighed but alternative drugs (e.g., vasopressin) may be more effective. That way, corticotherapy may improve vascular response to endogenous catecholamines and reduce the synthesis of nitric oxide (vasodilatator). Identification and precise characterization of sepsis subtypes are hoped to enable a predictive enrichment for corticotherapy [18]. However, COVID-19 has taught us that there are other indications for corticotherapy in severe infections besides refractory shock. Elucidating the GCs mechanism-of-action may point to novel indications of the low-dose corticotherapy in other critical conditions. A prime candidate appears to be a life-threatening hyperinflammation and/or endotheliopathy, in which GCs can be used as an adjunctive treatment in combination with other anti-inflammatory drugs. Specific sites of infection (the lungs, abdomen, urinary tract) can also modulate the GCs efficacy. An in-depth retrospective investigation of the clinical (hemodynamic, respiratory) and laboratory (inflammatory, metabolic, cell injury) effects of GCs in COVID-19 can unravel specific indications for corticotherapy applicable to other conditions.

### Are the currently recommended doses of dexamethasone in COVID-19 immunomodulatory?

Understanding of the immunomodulatory effects of dexamethasone in COVID-19 is not solely a research question; it may also help identifying drugs that synergize with this treatment. A single-cell RNA sequencing revealed that dexamethasone-treated critically ill COVID-19 patients downregulate expression of genes in B-cell, plasmablasts and some T-cell subsets [19]. Corticotherapy inhibited ROS generation in the circulating T-cells from the patients [20]. Moreover, patients receiving GCs had lower CD4^+^ counts and downregulated HLA-DR on circulating monocytes [21]. Dexamethasone treatment reduced circulating neutrophil counts, particularly the subset with IFN-activated signaling, while it increased the immature and immunosuppressive neutrophils [19]. Accordingly, plasma proteomics showed a suppression of calprotectin and neutrophil serine protease by GCs [19], both of which support a functional modulation of neutrophils. Importantly, the expression of the glucocorticoid receptor (GR)-encoding gene in the lung-infiltrating neutrophils was inversely correlated with COVID-19 severity [22]. A complementary hamster study showed an inhibition of the SARS-CoV-2-evoked neutrophil alterations in the lung by dexamethasone [23]. Dexamethasone also reduced neutrophilic pulmonary inflammation in the mouse model of COVID-19 and inhibited B-cell responses [24]. Overall, the current evidence suggests that steroids modulate neutrophilic, T-cell and B-cell mediated pulmonary inflammation in COVID-19, which in turn decrease endothelial and epithelial injury. Since GCs suppress the IFN-mediated signaling, it should be investigated whether corticotherapy impairs the beneficial antiviral IFN activity. It is speculative that higher GCs doses could be more protective for the pulmonary response. The emerging data on the immunomodulatory role of GCs in COVID-19 are in sharp contrast to the existing knowledge on the effects of similar hydrocortisone doses in septic shock. Single-cell sequencing techniques supported by a compartment-specific approaches are needed to explore the effects of steroids in septic shock.

## Know your patient

### How can we identify responders versus non-responders?

An underestimated problem in the ICU practice is GC resistance (GCR) – currently undetectable at the bedside. GCR is common in multiple diseases, such as rheumatoid arthritis, COPD, hyperinflated ARDS and sepsis [25]. An acquired GCR is considered as a pathological host response, because endogenous GCs ineffectively control inflammation, glucose metabolism and endothelial dysfunction [26]. It is urgent we find ways to identify GC responders with the goal of overcoming GCR. Several strategies for predictive enrichment are emerging aimed at pre-identification of responders by analysis of: (1) corticosteroid-response genes (e.g., GLCCI1, BHSD1) and specific signatures by transcriptomics [27], (2) dynamic markers directly assessing response to corticotherapy and (3) the inflammatory environment of the host [28]. The effects of GCs depend on the inflammatory status (GCs were effective only before the experimental TNF challenge [29] and in septic mice with high circulating IL-6 [30]). Also, in COVID-19 ARDS patients with the hyperinflammatory phenotype, the corticotherapy was more effective than in the hypoinflammatory patients [31]. Intriguingly, reanalysis of the hydrocortisone trial in sepsis showed that corticotherapy was harmful to patients with an immunocompetent transcriptomic signature but not in those with immunosuppression [32]. It is speculative whether overcoming of GCR brings more benefits in selected patients; currently, a GCs dose increase is the only available possibility to overcome this drug resistance. Conversely*,* GC overdosing can be harmful in general patients’ population as evidenced by e.g., the emergence of mucormycosis [33]. These points clearly show that better understanding of both the pathological processes in sepsis and precise action of corticosteroids is required for the development of personalized corticotherapy. Identification and operationalization of point-of care biomarkers rapidly characterizing1 the resistance/responsiveness to corticotherapy is urgently needed.

### When is the patient benefitting the most from corticotherapy?

**Fig. 2:**
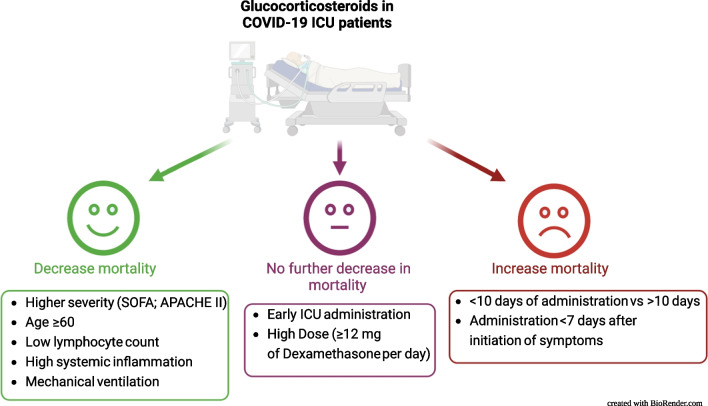
Clinical determinants of the effects of corticotherapy in ICU-treated COVID-19 patients

The results from the RECOVERY trial (subsequently reasserted by the WHO metanalysis) did not provide information on all patient subpopulations, thus, potential adverse-effects must be taken into account in any risk/benefit assessment [1, 34]. Currently, it remains unclear whether corticotherapy is similarly beneficial in aged patients compared to the younger ones. This is a valid question due to the immunosenescence which may profoundly impact the host response. Of note, the RECOVERY trial reported no efficacy of GCs in patients > 70 years. Another recently published study demonstrated that mortality rose in patients ≥ 80 years [35]. A recent large multicenter observational study in the ICU-admitted patients found a survival benefit in patients > 60 years [36]. The timing of GCs prescription is another valid question: an early administration represents other clinical scenarios that could modulate the benefits conferred by GCs. Remarkably, in the CIBERESUCICOVID study [36], it was revealed that GCs increased mortality in the ICU-admitted patients when the therapy was initiated < 7 days of symptoms onset. Regarding early GC administration in ICU patients, the results are controversial; an early administration seemed beneficial [37] but patients receiving GCs with a 48 h delay were not discarded [36]. It remains unclear what the most efficient doses are in specific clinical situations. It was clearly demonstrated that in COVID-19, 12 mg of dexamethasone was not more efficient (versus 6 mg), leading to the recommendation of the low dosage [36]. Duration of GC treatment also remains an open question. The results of a recent meta-analysis showed that treatment for more than 7 days improved survival compared to shorter treatments [38]. The GC treatment in the ICU patients for more than 10 days was associated with a higher reduction in 90-day mortality [36]. The clinical characteristics associated with the efficacy of GCs are summarized in **Fig. ****2**. Finally, a similar uncertainty is caused by a question whether dose-tapering can reduce a lung inflammatory rebound and reduce an extubation failure in COVID-19 ARDS patients.

### Can a positive response to GCs be boosted in septic and COVID-19 patients?

While much desired, such an effect is difficult to investigate. COVID-19 pandemic, however, has created a unique environment in which such studies can be pursued. Once the GCs became SoC in COVID-19, all RCTs tested any given intervention together with dexamethasone. Tocilizumab (IL-6 receptor-blocking monoclonal antibody) is such an example. Notably, only RCTs combining GC and tocilizumab were beneficial [39]. The biological mechanism responsible for this synergy remains elusive. It can be hypothesized that such a drug combination is also beneficial in sepsis as the IL-6 levels in COVID-19 patients are comparable and/or lower than in septic shock patients. Another promising anti-cytokine drug, anakinra (IL-1R antagonist) was shown to improve outcomes of COVID-19 patients with high circulating suPAR [40]. Similarly, the majority of anakinra-treated patients also received dexamethasone as SoC. Thus, it is unclear whether the benefits of anakinra were in any way potentiated by steroids. Given that neutrophils from steroid-treated patients showed an increased expression of IL-1R2 (a decoy receptor for IL-1) such a synergy is likely. The anti-cytokine therapies are especially attractive due to their specificity. It has been also suggested that the NLRP3 inflammasome inhibitors synergize with GCs to control the immunopathology of COVID-19, yet clinical data are missing. The Janus-associated tyrosine kinase -1, -2 inhibitor- baricitinib also seems to enhance the effects of GCs in COVID-19 [41]. Another potential approach increasing the response to GCs is an inhaled nitric oxide (NO). In porcine endotoxemia, NO was shown to increase the expression of GCs receptor and potentiate anti-inflammatory effects of GCs [42]. An alternative pathway relies on selective GR agonists and modulators favoring GR monomers and/or dimers [43]. Although purely speculative in COVID-19, the anti-inflammatory synergism between GCs and antithrombotic drugs has also been demonstrated in vitro [44]. In the context of viral infections, the potential interaction between GCs and recombinant interferons is of special interest.

## Know your drug and its targets

### What are the molecular and spatial dependencies that modulate effects of GCs in various cells and tissues?

Both natural steroids and synthetic GCs bind to the constitutively and ubiquitously expressed GR and thereby regulate up to 20% of existing gene families [45]. However, this regulation is not uniform and the spatial configuration of the GC-GR complexes (either homodimeric or monomeric) appears to determine their downstream action. The current understanding is that the GC-GR homodimer up-regulates gene expression directly as a transcription factor (TF) via so-called transactivation [46]. In contrast, the GC-GR monomer complex prevents other TFs (e.g., NF-κB) from binding to their target genes and represses gene expression in a process described as transrepression [46]. These various GC-GR configurations may often generate opposite effects. For example, homodimeric GC-GR configuration generally favors anti-inflammatory effect [45] and GR binding to DNA-bound STAT3 was shown to inhibit STAT3 gene expression, whereas STAT3 binding to DNA-bound GR resulted in transcriptional synergy [47]. Additionally, there are at least 8 alternative splicing variants of the GR and these GR isoforms differ significantly in their relative presence among cells; e.g., GRα-C was higher in the pancreas and lung compared to the liver [48]. This generates a unique cell-specific genomic signal by those individual isoforms (either induction or repression) that is independent from the gene pool commonly regulated by all isoforms (approx. 20%). In critical illness, the expression profiles of key regulators of local glucocorticoid action (e.g., GILZ, FKBP51) vary between tissues [49]. For example, there was strong expression difference of those regulators between circulating neutrophils (suppressed) and monocytes, whereas mouse sepsis indicated an unequal GC availability among tissues. Importantly, GCs can also exert their action via non-genomic signaling (e.g., membrane lipids properties, mitochondrial function) but its modulatory role in the GCs-dependent effects is virtually unknown.

### Can we make the GCs action more specific?

Administration of GCs, especially protracted, triggers adverse various effects. For example, corticotherapy in COVID-19 is related with an increased risk of nosocomial pneumonia [36]. Questions regarding (1) cell-type specific mechanisms, (2) induced GC receptor (GR) configuration and (3) required GC dosing in critically ill patients remain unanswered. Due to their broad mechanism-of-action, GCs are largely unspecific “dirty” drugs. The dynamics of the GR homo- versus dimerization (thereby varying target gene expression; see Q7) can be potentially utilized for a more targeted GC use. Considering the dynamics of cytokines released in sepsis, it should be realized that there is a complex interplay and feedback between GCs and cytokines; a term “*context-specific mechanism of GCs action”* has been proposed to describe this phenomenon [45]. The above-mentioned strategies combining GCs with other immunomodulators are a prime example that GCs effects depend on the inflammatory environment [29]. The current debate should therefore sensitize clinicians to monitoring the inflammatory status of patients before prescribing immunomodulators. A new approach to increase the specificity of GC activity is to generate an antibody–drug conjugate as shown in CD163 combined with dexamethasone. This drug targets activated macrophages and suppresses cytokine release after experimental LPS stimulation. This is a powerful strategy and the effect is 50-fold higher compared to an unbound dexamethasone [50]. The modification of synthetic GCs is another pharmacological method to reduce systematic side effects when administered locally or via inhalation. The development of more potent GCs by a stronger binding to GR is an alternative option. Potent GC application is not only useful for topical application but also for systemic use when a strong binding, “warhead” is desired. This can efficiently boost the GC-GR homodimer configuration to maximize anti-inflammatory effects [51].

### Does the GCs type matter for sepsis or COVID-19 outcomes?

Different doses and types of GCs have been tested in ARDS and sepsis, which confounds the published analyses. Clinically, the different GCs are often interchangeable, and the main difference regards the mineralocorticoid activity. However, experimental evidence indicates that GCs also differ in modulation of particular responses [52]. The COVID-19 pioneer trial RECOVERY tested dexamethasone at 6 mg/day, a choice that was not solidly justified. A theoretical pharmacological equivalence based on prednisone can be compared; 5 mg of prednisone corresponds to: 20 mg of hydrocortisone (× 4); 4 mg of methylprednisolone; 0.75 mg of dexamethasone (1/6). The COVID-19 SoC dexamethasone (6 mg/d) corresponds to 40 mg of prednisone (0.6 mg/kg/d of prednisone over 10 days) [2]. The efficacy of dexamethasone and methylprednisolone in COVID-19 was compared only in low-evidence studies and the results are ambiguous [53]. Furthermore, a pulse of methylprednisolone in addition to SoC dexamethasone was not beneficial in severe COVID-19 [54]. The pharmacokinetic of the GCs should also be considered, particularly in critical care. The longer the half-life, the higher the risk of secondary infections due to immunosuppression. Understanding the beneficial GCs mechanisms-of-action in COVID-19 could lead to identification of a most suitable GCs agent(s). The systemic type and doses of GCs were higher in reports on ARDS and sepsis compared to COVID-19 studies. However, further evidence is needed to elucidate optimal GCs dosing and selection thereof.

### How to effectively utilize novel- generation GCs?

The mechanism of ICU-acquired GCs resistance is complex, unsolved and depends on the inflammatory environment of the host [25]. At least several strategies to overcome unwanted side effects of GCs have been developed. A topical use of GCs is common in this context and the drug repertoire has been constantly growing. For example, a new non-steroidal GR agonist *LEO134310* has been recently characterized with an advantage of a rapid deactivation in the blood [55]. In addition, inhaled GCs have been SoC in asthma and COPD for many years. Interestingly, the local anti-inflammatory pulmonary effects are also beneficial in COVID-19 and prevent hospital admissions and death [56]. However, a trial comparing parenteral versus inhaled GCs in COVID-19 is missing. Other strategies aim at increasing GC specificity by an antibody binding [50] and association with long-circulating liposomes [45]. Moreover, modification of singular GC-induced proteins such as GILZ may also induce anti-inflammatory effects [45], albeit these strategies are still experimental. In contrast, *Fosdagrocorat* is a selective GR Dissociated Glucocorticoid Receptor Agonist (SEGRA) beneficial in rheumatoid arthritis [57]. The development of SEGRAs is based on a paradigm assuming that the GR conformation favors transcriptional (homodimer) or transactivational (monomer) effects [43]. A potential beneficial anti-inflammatory effect of selective GR agonists is an appealing option which combines an effective suppression of the host-response and simultaneous reduction in negative effects [43].

## Conclusion

Potent biological actions of GCs continue to position them as attractive drugs in critical care. The tremendous research effort during COVID-19 pandemic has established a clear new indication for GCs and has shed some light on their mechanism-of-action. A better understanding of the heterogeneity of the critical illness syndromes, the mode of GCs actions and employment of the precision medicine concepts in the ICU, will further enhance the overall utility of corticotherapy in critical care. The ten questions we postulated above aim at paving the road toward that achievable goal (Table 1).

**Table 1:** Ten urging questions about glucocorticosteroids (GCs) in critical care and proposed studies needed to answer them

Question	Needed studies
Q1: Why are GCs effective in COVID-19 but not in bacterial sepsis nor severe influenza?	1. Molecular characterization of the systemic vs local response to corticotherapy in COVID-19, influenza, sepsis 2. Longitudinal study of inflammatory response in presence/absence of steroids
Q2: Is refractory vasoplegia the only viable target for GCs treatment in septic shock?	1. Efficacy of corticotherapy in specific endotypes of sepsis 2. Retrospective analysis of clinical and laboratory effects of corticotherapy in COVID-19
Q3: Are the currently recommended doses of dexamethasone in COVID-19 immunomodulatory?	1. Influence of GCs on the IFN antiviral response 2. Single-cell resolution and local immunity focused studies on the effects of GCs in sepsis
Q4: How can we identify responders versus non-responders?	1. Development of rapid and point-of care available biomarkers of corticotherapy resistance/responsiveness
Q5: When is the patient benefitting the most from corticotherapy?	1. Initiation time of the corticotherapy on the clinical effect 2. Optimization of dose-tapering in various patient cohorts
Q6: Can a positive response to GCs be boosted in septic and COVID-19 patients?	1. Clinical studies of corticotherapy combined with other immunomodulators in sepsis (similarly to COVID-19) 2. Identification of drugs increasing sensitivity to GCs
Q7: What are the molecular and spatial dependencies that modulate effects of GCs in various cells and tissues?	1. Organ-and cell-specific distribution of the GC receptor complexes 2. Non-genomic effects of GCs in sepsis
Q8: Can we make the GCs action more specific?	1. Evaluation of context-specific action of GCs in septic patients 2. Identification of the relevant cell target in sepsis to test specific antibody-GC conjugate
Q9: Does the GCs type matter for sepsis and COVID-19 outcomes?	1. Comparison of the biological effect of different GC preparations in septic shock and COVID-19 2. Pharmacokinetic studies of GCs in septic shock
Q10: How to effectively utilize novel- generation GCs?	1. Evaluation of the novel short-acting GC receptor agonists and liposomal formulations in severe infections 2. Comparison of parental versus inhaled corticotherapy in pulmonary infections

## Data Availability

Not applicable.
